# Isolation and Molecular Identification of Keratinophilic Fungi from Public Parks Soil in Shiraz, Iran

**DOI:** 10.1155/2013/619576

**Published:** 2013-07-15

**Authors:** Keyvan Pakshir, Moosa Rahimi Ghiasi, Kamiar Zomorodian, Ali Reza Gharavi

**Affiliations:** Department of Parasitology and Mycology, Basic Sciences in Infectious Diseases Research Center, School of Medicine, Shiraz University of Medical Sciences, Shiraz 71345-45794, Iran

## Abstract

*Introduction*. Keratinophilic fungi are an important group of fungi that live in soil. The aim of this study was to isolate and identify keratinophilic fungi from the soil of different parks in Shiraz. *Materials and Methods*. A total of 196 soil samples from 43 parks were collected. Isolation of the fungi was performed by hair bait technique. The isolated colonies were identified by morphologic feature of macro- and microconidia and molecular method, using DNA sequence analysis. ITS region of ribosomal DNA was amplified and the PCR products were sequenced. *Results*. 411 isolates from 22 genera were identified. *Fusarium* (23.8%), *Chrysosporium* (13.13%), *Acremonium* (12.65%), *Penicillium* (12.39%), *Microsporum gypseum* (1.94%), *Bionectria ochroleuca* (1.21%), *Bipolaris spicifera* (1.21%), *Scedosporium apiospermum* (0.82%), *Phialophora reptans* (0.82%), *Cephalosporium curtipes* (0.49%), *Scedosporium dehoogii* (0.24%), *Ochroconis constricta* (0.24%), *Nectria mauritiicola* (0.49%), *Chaetomium* (0.49%), *Scopulariopsis* (0.24%), *Malbranchea* (0.24%), and *Tritirachium* (0.24%) were the most important isolates. Most of the fungi were isolated from the soils with the PH range of 7 to 8. *Conclusion*. Our study results showed that many keratinophilic fungi isolated from the parks soil are important for public health and children are an important group at a high risk of being exposed to these fungi.

## 1. Introduction

Keratinophilic fungi are ecologically an important group of fungi which could be found in soil [1]. Some groups of these fungi are causative agents of cutaneous fungal infections named dermatophytosis, and the other saprophyte fungi mainly represent hyalohyphomycosis [2, 3]. The prevalence of these fungi depends on different factors, such as the presence of creatinine in the soil, pH, and geographical location [1]. Some of these fungi, such as dermatophytes, are well known to cause tinea infections which could be transmitted from soil to humans. In general, soil could be considered as a reservoir for human infection. Forests, farmyards, park soils, and sediments of the rivers and oceans containing humus and organic materials are the best candidates for growth of keratinolytic and saprophytic fungi [4].

During the past years, many researchers reported about isolation of keratinophilic fungi around the world [5–14]. Also, a lot of reports were available about the isolation of geophilic dermatophytes and keratinophilic fungi from the soils of many parts of Iran and also dermatophytosis due to the geophilic fungi during the last decade [15–18]. Nowadays, most people spend their time with their children in the parks for fun and are potentially at risk for direct contact with soil and being exposed to keratinophilic fungi [4]. Shiraz is one of the most populous cities of Iran and the capital of Fars province which is located in the southwest of Iran. It has a moderate to warm climate (−3 to 40°C) with rain falling about 350 mm/year, land area of approximately 225 km^2^, and more than one million population. Up to now, there were no data about keratinophilic fungi in soil of Shiraz and no reports about the molecular identification of these fungi around Iran. Thus, the present study aimed to isolate and identify keratinophilic fungi from soil of the popular parks in Shiraz by molecular and morphological analysis. 

## 2. Materials and Methods 

### 2.1. Sample Collection

In this descriptive study, 196 soil samples were collected from various sites of 43 different parks around Shiraz during spring 2011. The samples were collected from the superficial layer of the soil whose depth did not exceed 5–10 cm by using an iron spatula. In doing so, 300–400 gram of soil was collected in sterile polyethylene bags and brought to laboratory for further processing.

### 2.2. Measuring of Soil pH

PH of each soil sample was measured after preparation of soil suspension (one gram of soil to five mL deionized water) using pH meter [17, 19].

### 2.3. Isolation and Identification of the Isolates

We used Vanbreuseghem's hair bait technique for isolation of keratinophilic fungi [20]. Briefly, each soil sample was thoroughly mixed, and about 70 gram of the soil was packed in a sterile 90 mm Petri dish. Then, several pieces of sterile healthy children hair fragments were dispersed over the surface of the soil samples and moistened with sterile distilled water supplemented with antibiotic solutions, chloramphenicol (0.2 g/mL), and strepto-penicillin (1000 IU/mL). All the baited soil Petri dishes were incubated at room temperature (20–25°C) in the dark for 2 months and got moistened if necessary. After observation of colony growth around the hairs, the colonies were subcultured on Sabouraud's dextrose agar (SDA) with and without chloramphenicol (50 mg/L) and cycloheximide (500 mg/L) and purified. The fungi were identified based on the conventional method (colony morphology and macro- and microconidia characteristics) and DNA-based identification techniques. 

### 2.4. DNA-Based Identification Techniques (DNA Sequence Analysis)

Molecular identification of the unknown isolates was achieved by DNA sequence analysis. First, the fungi were grown in flasks containing Sabouraud's dextrose broth and incubated at 25°C for several days using shaker rotator. After colony growth, the culture was filtered (Millipore, USA) and the fungal mass was washed with distilled water for several times and stored in a freezer (−20°C) for further processing. 

DNA was extracted by using Lee technique [21] with mild modification. First, the frozen mycelium mass was smashed by mechanical pressure using sterile pounder and liquid nitrogen. The acquired powder was then mixed with lyses buffer and the DNA was extracted. The ITS1–5.8S–ITS 2 rDNA was amplified using ITS1 and ITS4 as forward and reverse primers as described by White et al. [22]. Amplification was performed in 50 *μ*L reaction volumes containing 5 *μ*L of 10× buffer, MgCl_2_ (25 mm) 1.5 *μ*L, dNTP (10 mM) 0.5 *μ*L, 0.5 *μ*L of each 0.2 Mm primer (ITS1: 3′-TCC-GTA-GGT-GAA-CCT-GCG-G-5′ and ITS4: 3′-TCC-TCC-GCT-TAT-TGA-TAT-GC-5′), Taq Polymerase (1.25 U) 0.5 *μ*L, DNA sample 1 *μ*L, and distilled water 40.5 *μ*L. The PCR reaction was carried out using a Thermal Cycler (R corbet cg1-96) with the following conditions: denaturation at 94°C for 5 min, 34 cycles of (30 s at 94°C, 45 s at 56°C, and 45 s at 72°C) extension at 72°C for 7 min, and storage at 4°C. Negative controls were also used in each set of reactions. The final products were analyzed by electrophoresis on 1.2% agarose gel (Sigma) and stained with 0.5 *μ*g mL^−1^ ethidium bromide. In addition, the PCR products were sent for sequencing in both directions (Bioneer, Korea). The sequence results were processed by using the web-based blasting program, basic local alignment search tool (BLAST), at the NCBI site (http://www.ncbi.nlm.nih.gov/BLAST), and the data were compared with the NCBI/Genebank database [23].

### 2.5. Data Analysis

The study data were analyzed through Fisher exact test and Chi-square test. 

## 3. Results

**Figure 1:**
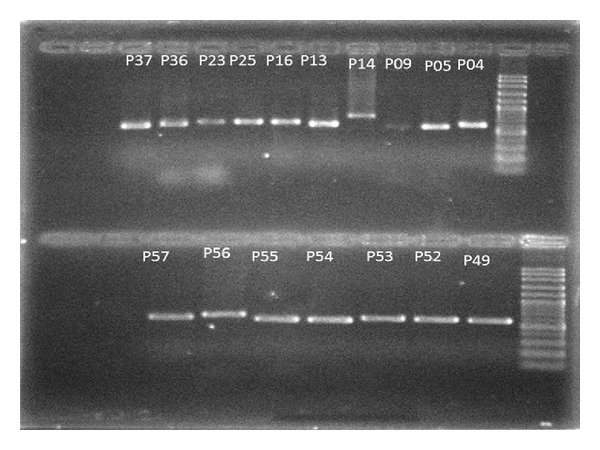
Agarose gel electrophoresis and PCR products bands; lines 1 and 2 (marker), lines 3–12 (up), and 2–8 (down) are PCR products of many unknown fungi.

PCR products bands on gel agarose are presented in Figure 1. From the 196 soil samples, a total of 411 colonies of keratinophilic fungi were isolated from 43 parks. The fungal isolates belonged to 22 genera as follows: *Fusarium *(25.30%), *Penicillium* (13.13%), *Chrysosporium *(13.13%), *Acremonium *(12.65%), *Aspergillus* (11.92%), *Mucor* (9.48%), *Paecilomyces* (4.13%), *Microsporum* (2.42%), *Bipolaris* (1.45%), *Bionectria* (1.21%), *Pseudallescheria *(0.73%), *Phialophora *(0.73%), *Alternaria* (0.73%), *Nectria* (0.48%), *Cephalosporium *(0.48%), *Chaetomium *(0.48%), *Scopulariopsis* (0.24%), *Scedosporium* (0.24%), *Verticillium* (0.24%), *Malbranchea *(0.24%), *Tritirachium *(0.24%), and *Ochroconis* (0.24%). More details about the species are presented in Table 1. 

*Fusarium *spp. was the most common fungal isolate among the parks. Besides, eight species of *Microsporum gypseum *were isolated from four parks with soil pH between seven and nine and two species of *Microsporum fulvum* from one park with the same pH. Most of the fungi were isolated from the soil samples with pH between 7 and 8 (66.42%), and no colony growth was seen in pH > 9 as shown in Table 2. The study results revealed no significant correlation between soil pH and fungal species. More details about the isolates and soil pH are presented in Table 2.

## 4. Discussion

The keratinolytic activity of keratinophilic fungi is important for ecology and has attracted many researchers' attention around the world. Keratinophilic fungi play an important role in the natural degradation of keratinized residues in the soil [24, 25]. Some types of these fungi, such as geophilic dermatophytes, live in soil and could be transmitted to humans as well as animals and cause cutaneous fungal infections [26, 27]. Parks are among the popular public places for the people to spend their time and have fun with their family. Our study revealed the presence of keratinophilic fungi, such as dermatophytes, in the soil of Shiraz parks. *M. gypseum* is a frequent geophilic dermatophyte commonly distributed in soil worldwide. Although nondermatophyte fungi isolates were more common than dermatophytes in the present study, *M. gypseum* and *M. fulvum* were the main dermatophytes which were isolated from four parks. These parks potentially have a high risk for transmission of fungal infections. Previously, these fungi were isolated from the soil samples of different parts of Iran [28–31].

Some species of *Aspergillus*, *Fusarium solani*, and *Bipolaris spicifera* are the causative agents of mycotic keratitis [32]. Our study showed that the genus *Fusarium* was the first dominant fungus in the soil of Shiraz, which is in agreement with the other studies conducted on the issue. Moallaei et al. [16, 30] also reported that *Fusarium* was the most prevalent saprophyte in South and Razavi Khorasan Provinces. The second most common species isolates in our study were *Chrysosporium* and *Penicillium*. *Chrysosporium* species have been reported to be the causative agents of disseminated diseases [33]. *Chrysosporium tropicum* was reported from comb lesion in two different breeds of chicken in India [34]. 

In this study, molecular method was utilized for identification of keratinophilic fungi for the first time in Iran. We could isolate and identify some genera of fungi, such as* Bionectria *spp., which is important in natural products and medicine. The genus *Bionectria* is endophytic and has great potential for medicinal and agricultural applications [35].

*Bionectria* species are known as a destructive mycoparasite and grow inside the fungal host hyphae. They are used as a biocontrol agent of plant-pathogenic fungi and are infrequently isolated from dead insects. Besides, they are known as a parasite of living nematodes, ticks, and myxomycetes [35, 36]. 

*Tritirachium* species are an insect pathogen whose natural habitats are soil and decaying plant materials. These fungi are occasionally isolated from head and nail infections [37, 38]. Furthermore, *Ochroconis *spp. are dematiaceous fungi that cause deep mycoses, such as chromomycosis, around the world. There are many reports regarding the fungal diseases caused by this fungus [39–41].

*Scedosporium apiospermum *is a common soil fungus with a worldwide distribution. Environmental isolations have been made from sewage sludge, polluted streams, and manure of poultry and cattle. In Australia, Cooley et al. [42] reported 59 cases of scedosporiosis caused by *Scedosporium apiospermum* and *S. prolificans* in patients with underlying diseases. Invasive infections in normal patients are usually caused by traumatic implantation. So, fungal infections could acquire or spread through playgrounds in parks.

The current study investigated the relationship between the frequency of fungi and the soils pH. Böhme and Ziegler [19] reported the effect of the soil pH on the presence of keratinophilic fungi for the first time. Many researchers stated that keratinophilic fungi could not be found in the soils with low pH levels (3–4.5). Garg et al. [3] also reported that the acidic soils with pH = 5.9 were free of keratinophilic fungi. Moreover, Asahi et al. [43] demonstrated that keratinolytic enzymes were produced in pH of 6–9, and particularly the extracellular keratinase was active in pH = 9. In the present study, all the 411 keratinophilic fungi were isolated from the soils with pH between 6 and 9. We found 66.42%, 32.6%, and 0.97% keratinophilic fungi from the soil samples with pH of 7.01–8, 8.01–9, and 6–7, respectively. These findings have been confirmed by other studies as well. Overall, in this study, 47% of the places were contaminated with keratinophilic fungi and geophilic dermatophyte species isolated from the soils of four parks. These areas potentially have a high risk for causing cutaneous fungal infections in humans and animals and could be considered as a source of these infections.

## Figures and Tables

**Table 1:** Frequency of keratinophilic fungi isolated from soils with different PH.

Fungal genus	pH							
	6-7		7-8		8-9		>9	
	*n*	%	*n*	%	*n*	%	*n*	%
*Aspergillus *	0	0	31	11.35	18	13.43	0	0
*Acremonium *	0	0	35	12.82	17	12.68	0	0
*Alternaria *	0	0	2	0.73	1	0.75	0	0
*Scopulariopsis *	0	0	1	0.36	0	0	0	0
*Ochroconis *	0	0	1	0.36	0	0	0	0
*Bipolaris *	0	0	4	1.46	2	1.49	0	0
*Bionectria *	0	0	4	1.46	1	0.75	0	0
*Cephalosporium *	0	0	1	0.36	1	0.75	0	0
*Paecilomyces *	2	50	7	2.56	8	5.97	0	0
*Scedosporium *	0	0	4	1.45	0	0	0	0
*Tritirachium *	0	0	1	0.36	0	0	0	0
*Penicillium *	2	50	36	13.1	16	11.94	0	0
*Fusarium *	0	0	66	24.1	38	28.35	0	0
*Phialophora *	0	0	2	0.73	1	0.75	0	0
*Chrysosporium *	0	0	39	14.28	15	11.19	0	0
*Chaetomium *	0	0	2	0.73	0	0	0	0
*Mucor *	0	0	22	8.42	16	11.94	0	0
*Microsporum *	0	0	8	3.66	0	0	0	0
*Malbranchea *	0	0	1	0.36	0	0	0	0
*Nectria *	0	0	2	0.73	0	0	0	0
*Verticillium *	0	0	1	0.36	0	0	0	0

Total	4	100	273	100	134	100	0	0

**Table 2:** Distribution frequency of keratinophilic fungi isolated from Shiraz parks soil.

Species	Number	Percent
*Fusarium* spp.	95	23.08
*Fusarium chlamydosporum *	4	0.97
*Fusarium oxysporum *	3	0.82
*Fusarium solani *	2	0.49
*Chrysosporium* spp.	54	13.13
*Acremonium* spp.	52	12.65
*Penicillium* spp.	51	12.39
*Penicillium crustosum *	2	0.49
*Penicillium palmae *	1	0.24
*Aspergillus niger *	40	9.73
*Aspergillus fumigatus *	6	1.45
*Aspergillus sclerotiorum *	2	0.49
*Aspergillus flavus *	1	0.24
*Mucor* spp.	39	9.48
*Paecilomyces* spp.	17	4.13
*Microsporum gypseum *	8	1.94
*Microsporum fulvum *	2	0.49
*Bipolaris spicifera *	5	1.21
*Bipolaris* sp.	1	0.24
*Bionectria ochroleuca *	5	1.21
*Scedosporium apiospermum *	3	0.82
*Scedosporium dehoogii *	1	0.24
*Phialophora reptans *	3	0.82
*Alternaria solani *	2	0.49
*Alternaria alternata *	1	0.24
*Cephalosporium curtipes *	2	0.49
*Chaetomium* spp.	2	0.49
*Nectria mauritiicola *	2	0.49
*Scopulariopsis* sp.	1	0.24
*Verticillium* sp.	1	0.24
*Malbranchea* sp.	1	0.24
*Tritirachium* sp.	1	0.24
*Ochroconis constricta *	1	0.24

Total	411	100%
